# Regulation of semaphorin 4D expression and cell proliferation of ovarian cancer by ERalpha and ERbeta

**DOI:** 10.1590/1414-431X20166057

**Published:** 2017-02-20

**Authors:** Y. Liu, Y. Hou, L. Ma, C. Sun, J. Pan, Y. Yang, H. Zhou, J. Zhang

**Affiliations:** 1 Department of Gynecology, the Second Affiliated Hospital, Kunming Medical University, Kunming, Yunnan, China; 2 Department of Reproduction and Genetics, the Second Affiliated Hospital, Kunming Medical University, Kunming, Yunnan, China; 3 Department of General Surgery, the Second Affiliated Hospital, Kunming Medical University, Kunming, Yunnan, China

**Keywords:** Semaphorin 4D expression, ERα, ERβ, Ovarian cancer, Cell proliferation

## Abstract

Ovarian cancer is one of the most common malignancies in women. Semaphorin 4D (sema 4D) is involved in the progress of multiple cancers. In the presence of estrogen-like ligands, estrogen receptors (ERα and ERβ) participate in the progress of breast and ovarian cancers by transcriptional regulation. The aim of the study was to investigate the role of sema 4D and elucidate the regulatory pattern of ERα and ERβ on sema 4D expression in ovarian cancers. Sema 4D levels were up-regulated in ovarian cancer SKOV-3 cells. Patients with malignant ovarian cancers had significantly higher sema 4D levels than controls, suggesting an oncogene role of sema 4D in ovarian cancer. ERα expressions were up-regulated in SKOV-3 cells compared with normal ovarian IOSE80 epithelial cells. Conversely, down-regulation of ERβ was observed in SKOV-3 cells. Forced over-expression of ERα and ERβ in SKOV-3 cells was manipulated to establish ERα^+^ and ERβ^+^ SKOV-3 cell lines. Incubation of ERα^+^ SKOV-3 cells with ERs agonist 17β-estradiol (E2) significantly enhanced sema 4D expression and rate of cell proliferation. Incubated with E2, ERβ^+^ SKOV-3 cells showed lower sema 4D expression and cell proliferation. Blocking ERα and ERβ activities with ICI182-780 inhibitor, sema 4D expressions and cell proliferation of ERα^+^ and ERβ^+^ SKOV-3 cells were recovered to control levels. Taken together, the data showed that sema 4D expression was positively correlated with the progress of ovarian cancer. ERα positively regulated sema 4D expression and accelerated cell proliferation. ERβ negatively regulated sema 4D expression and inhibited cell multiplication.

## Introduction

Ovarian cancer is the most common cause of death in women with gynecological malignancies, and it has the highest mortality rate among women in the world. Because of the lack of definitive early symptoms and efficient, specific and sensitive markers for ovarian cancer monitoring, the majority of patients diagnosed with late-stage ovarian cancer typically have a 5-year survival of 30% (1,2). Prevention and cure of ovarian cancer is still a great challenge.

However, solid evidence has established that steroid-resembling estrogen is related to increased breast cancer risk (3–5). Steroid estrogen analogues are still the most common drugs used to relieve the menopausal symptoms of women in menopausal hormone therapy (MHT) (6,7). However, emerging data in recent years suggest that estrogen is also implicated in the progression of ovarian cancer. Epidemiological studies reveal that long-term use of estrogen-only hormone replacement therapy increases a woman's risk of ovarian cancer (8,9). Utilization of anti-estrogen intervention inhibits the growth of ovarian carcinoma *in vitro* and *in vivo* (10,11). Data from a rat model demonstrated the growth promoting effects of estrogen on ovarian tumors in mice (12,13). Estrogen plays a physiological role through estrogen receptors (ERs) mediation. There are two ERs isoforms, ERα and ERβ, which are members of the nuclear receptors superfamily of ligand-dependent transcription factors (14). Although ERα and ERβ have structural and functional homologies, they may regulate the same genes in opposite ways, following a yin-yang hypothesis (15,16). In general, ERα is seen as an oncogene by promoting gene expression related to survival and proliferation of cancer cells. Whereas ERβ is usually described as tumor suppressor by having anti-proliferation and pro-apoptosis effects. The role of ERα in breast cancer have been well established, but the ER's role in ovarian cancer is still relatively vague by comparison (17).

The semaphorins family are grouped into eight subclasses and contain more than 30 members, which were initially identified as constituents of the nervous system complex that direct the growing axons to their targets (18). However, recent evidence indicates that expression levels of certain members of semaphorins, such as semaphorin 4D, semaphorin 5C, and semaphorin 6B etc., were altered in a variety of cancer cells. These semaphorins promoted tumor angiogenesis and increased tumor progression (19–21). Semaphorin 4D, a member of class 4 semaphorins and also known as CD 100, has been shown to be up-regulated in aggressive cervical, head and neck, prostate, colon, breast, and lung cancers (22). Under the interaction with its receptor plexin B, sema 4D facilitated the growth, invasion, and migration of cancer cells, promoting carcinogenesis and metastasis (23). Although many genes have been identified to be sema 4D-regulated targets in cancer cells, the up-stream regulatory mechanism of sema 4D is rarely explored (22). Whether sema 4D involvement in the progress of ovarian cancer is regulated by ERα and ERβ is still unclear.

In the present study, we detected the expression of sema 4D, ERα, and ERβ in ovarian cancer tissues and cells.

## Material and Methods

### Cells and regents

DMEM medium was purchased from Hyclone (SH30243.01B, USA). RNase, DNase, and DNA marker (Takara) were purchased from Shanghai Bito Co. Ltd., China. Methanol, haematine, ICI182-780 (ERs inhibitor), 17β-estradiol (E2), and eosin were purchased from Sigma Co. (USA). Sixty normal ovarian tissues, 60 benign ovarian cancer tissues, and 60 malignant ovarian cancer tissues were obtained from Second Affiliated Hospital of Kunming Medical University. The malignancies were classified into early phase (I-II) and terminal stage (III-IV) on the basis of surgical-pathologic staging FIGO, 2006. All ovarian cancer tissues were histologically confirmed by two pathologists. Patients did not receive medication before surgery. The collection of human tissue samples was approved and supervised by the Ethics Committee of Kunming Medical University. Human ovarian cancer SKOV-3 cells and human normal ovarian IOSE80 epithelial cells were purchased from Yingrun Biological Co. Ltd., China. 293TA cell line was purchased from Funeng Biological Co. Ltd., China.

### Cell culture

SKOV-3 and IOSE80 cell lines were cultured in RPMI 1640 medium supplemented with 10% (v/v) fetal bovine serum (FBS, HyClone). 293T cell lines were cultured in Dulbecco's Modified Eagle Medium (DMEM, HyClone) containing 10% (v/v) FBS, 100 units/mL of penicillin-streptomycin (Invitrogen, USA), and HOSE (Pricells, China). All cell lines were cultured in a humidified incubator in an atmosphere of 5% CO_2_ and 95% air at 37°C.

### Real-time (RT) PCR

Total RNA was isolated using Trizol (Invitrogen) according to the manufacturer's instructions. QRT-PCR was performed as previously described to assess the expression levels of sema 4D, ERα and ERβ using the 2^-ΔΔCT^ method (24). β-actin snRNA was used as internal standard to normalize the expression.

### Lentivirus packaging and stable cell lines establishment

ERα and ERβ highly expressed vectors were amplified from human genomic DNA and cloned into the XHO I and EcoRV site of the lentiviral vector pEZ-Lv105 (EX-A0322-Lv105, GeneCopoeia™). Viruses were packaged in 293T cells to generate 293T-pLV-ERα and 293T-pLV-ERβ lentiviral vector. SKOV-3 cells were cultured in 1640 medium with 10% FBS in a 37°C incubator with 5% CO_2_. 293T-pLV-ERα, 293T-pLV-ERβ or blank plasmid were co-transfected SKOV-3 cells with Lenti-Pac HIV Expression Packaging Kit following the manufacturer's instruction (GeneCopoeia™). Forty-eight hours after, transfection efficacy was evaluated by inverted fluorescence microscope. The supernatant was harvested, filtered and cleared by centrifugation at 500 *g* for 10 min at 4°C. Three days after infection, 2 μg/mL puromycin was added to the culture media to select the cell populations infected with the lentivirus for 2 weeks. The expression of ERα or ERβ was detected by RT-PCR and western blotting in these three cell lines as described above. The cell lines transfected with 293T-pLV-ERα and 293T-pLV-ERβ and stably expressing ERα and ERβ were named ERα^+^ and ERβ^+^ SKOV-3 cells, respectively. Cells transfected with blank plasmid were named control (CK) SKOV-3 cells.

### Immunohistochemistry

Tissue samples were fixed in PBS containing 4% paraformaldehyde. The slide was deparaffinized in dimethylbenzene followed by rehydration in 80% ethanol. Then, 3% hydrogen peroxide solutions were added to the tissue slides to quench the endogenous peroxidase. After washing with the PBS three times, the slides were incubated with anti-sema 4D antibody (Cat. #3134-1, Lot #2203119, Abcam, UK) overnight at 4°C, then secondary antibody (Dako Co., Denmark) were added and maintained for 2 h at room temperature. Finally, the slides were developed with 3,3-diaminobenzidine (DAB substrate kit for peroxidase; Vector Laboratories, China) and counterstained with hematoxylin. Images were obtained using an Aperio Scanscope in five randomized visual fields (Aperio Technologies, USA). Immunoreactivity for sema 4D was evaluated according to the numbers of positive cells and intensity of stained cells (21). The results were evaluated separately by two pathologists.

### HE staining

Tissue samples were fixed in PBS containing 4% paraformaldehyde. Then, the sections were deparaffinized in xylene and successively rehydrated with washes in 100, 95, 85, and 70% ethanol (2 min each). After that, slides were stained with hematoxylin (2 min) and rinsed with distilled water and 0.1% hydrochloric acid in 70% ethanol. The slide was then stained with 0.5% eosin for 2 min and rinsed again with distilled water. Finally, the slides were dehydrated with 95 and 100% ethanol successively, followed by xylene (3 min) and mounted with coverslips.

### Incubation of E2 or ICI182-780

ERα^+^, ERβ^+^, and CK SKOV-3 cell lines were cultured in 6-well plates containing PRMI 1640 medium. When the density of cells was appropriately 80%, 2 mL E2 solution (10^-6^ M, final concentration) were added to each well. After 24 h culture, the cells were harvested for sema 4D detection. For the ICI182-780 inhibitor treatment, a 100 nM ICI182-780 solution, which has been previously proved to disable ERs signaling efficiently, was added to the three cell lines cultured in PRMI 1640 medium in 6-well plates for 6 h (25,26). After that, 2 mL E2 solutions (10^-6^ M, final concentration) were added to each well and cultured for another 24 h.

### Cell proliferation assay

ERα^+^, ERβ^+^, and CK SKOV-3 cell lines with or without 100 nM ICI182-780 pre-incubation were seeded in a 96-well dish at a density of 5×10^3^ cells per well and incubated in 1640 medium containing 10% FBS and 10^–6^ M E2. After 0, 6, 12, 18, 24, and 30 h, the cells were washed with PBS and incubated in 100 µL 1640 medium containing 10 µL Cell Counting Kit-8 (CCK-8; Dojindo, Japan) solution for 120 min. The absorbance of each well at a wavelength of 450 nm was measured. Five duplicate wells were used for each measurement and experiments were repeated three times.

### Western blotting

The cells were lysed with RIPA lysis solution (DSL, USA). After total proteins were extracted, a BCA protein assay kit (Pierce, USA) was used to quantify the proteins. Equal protein amounts were mixed with the 4× loading buffer (Beyotime, China) and then boiled for 5 min for protein denaturation. A total of 15 μg protein from each sample was loaded for 12% SDS–PAGE gel electrophoresis and then transferred to a polyvinylidene fluoride (0.45 μm, PVDF) membrane. Then, the membrane was incubated with Ponceau S staining solution for 2 min to judge the transfer efficiency of proteins. Once the proteins were proven to have transferred to the membrane successfully, the membrane was incubated with 5% fat-free milk for 30 min. Then, the membrane was incubated with anti-sema 4D, ERα, ERβ (1:200, Santa Cruz, USA) or anti-β-actin (1:5000, NeoBioscience, China) antibodies at 4°C overnight. Finally, the membrane was washed and incubated with corresponding HRP conjugated-secondary antibodies at room temperature for 2 h. The bands were visualized using an enhanced chemiluminescence system (ECL, USA).

### Statistical analyses

Data are reported as means±SE. The statistical significance of differences between the groups was assessed by Student's *t*-test or, when more than two groups were compared, by one-way ANOVA, followed by Tukey's *post hoc* test. P<0.05 was considered to be significant.

## Results

**Figure 1:**
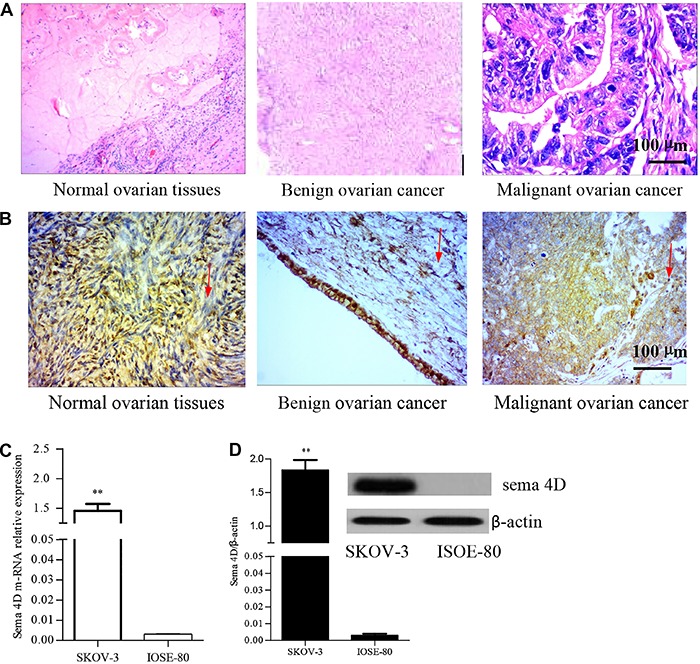
Semaphorin 4D (sema 4D) was up-regulated in ovarian tissues and cells. *A*, HE staining. *B*, sema 4D protein expression levels in ovarian cancer and normal ovarian tissues by immunohistochemistry. The arrows indicate sites of sema 4D expression. *C*, sema 4D m-RNA level using RT-PCR. Cells were harvested and total RNA was extract after 72 h culture. *D*, sema 4D protein level by western blotting. β-actin was used as an internal standard. SKOV-3: human ovarian cancer cells; IOSE-80: human normal ovarian cells. The experiments were repeated three times. Data are reported as means±SD (**P<0.01, *t*-test).

Sema 4D was highly expressed in ovarian cancer cells and tissues. To confirm the role of sema 4D in ovarian cancer, a total of 180 ovary samples including 60 benign ovarian cancer, malignant ovarian cancer and normal ovarian tissues each, were collected for the analysis of sema 4D protein expression by immunohistochemistry. These ovary samples were pathologically confirmed by HE staining (Figure 1A). The results showed that expression of sema 4D protein in benign ovarian cancer tissues and malignant ovarian cancer tissues was 58.3% (35/60) and 90.2% (55/60), respectively. In contrast, the expressive proportion (33.3%, 20/60) of sema 4D proteins in normal ovarian tissues was significantly lower than those in benign and malignant ovarian cancer tissues. Malignant issues classified as early pathological stage had a lower proportion of sema 4D protein expression than those classified as late stage (Figure 1B and Table 1), although values were not statistically significant. Meanwhile, sema 4D m-RNA and expression levels in ovarian cancer SKOV-3 cells were both found to be significantly higher than those in ISOE-80 cells (Figure 1C and D). Those observations uniformly confirmed the fact that sema 4D was up-regulated in ovarian cancer cells and tissues.

**Table:**
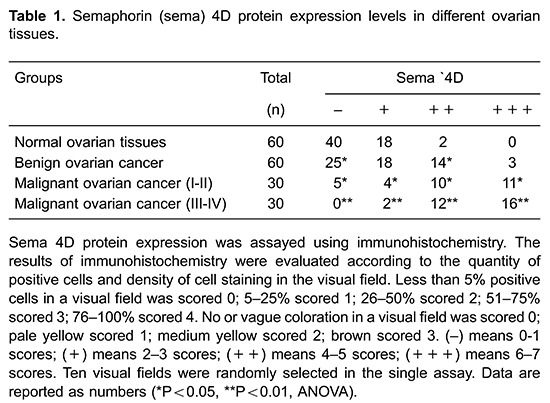

### ERα and ERβ were differentially expressed in SKOV-3 and ISOE-80 cells

**Figure 2:**
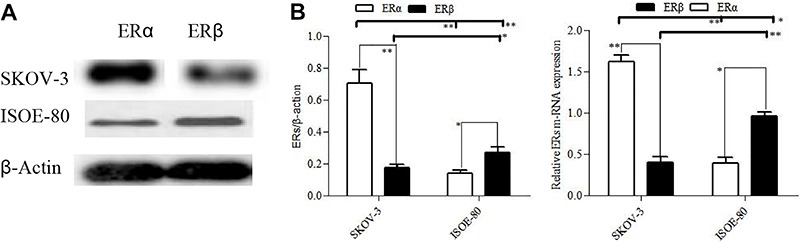
Estrogen receptor (ER)α and ERβ were conversely expressed in SKOV-3 human ovarian cancer cells and ISOE-80 human normal cancer cells. *A*, sema 4D protein expression by western blotting. *B*, Semaphorin 4D (sema 4D) m-RNA expression by RT PCR. β-actin was used as the internal standard. Data are reported as means±SD. (*P<0.05, **P<0.01, ANOVA).

As shown in Figure 2A and B, ISOE-80 cells have a higher ERβ expression than ERα both in nuclear acid and protein levels. The approximate ratio of ERα/ERβ was respectively 1/2.3 and 1/1.7 in nuclear acid and protein levels in ISOE-80 cells. Whereas the relative ERα m-RNA expression in SKOV-3 cells was significantly enhanced compared with those in ISOE-80. In contrast, a reduced ERβ m-RNA level in SKOV-3 cells was detected, resulting in the increased ratio of ERα:ERβ (3.3/1) in m-RNA levels. Correspondingly, ERβ protein expression was found to be down-regulated accompanied by an up-regulation of ERα protein expression in SKOV-3 cells. The expressive partners of ERα and ERβ were differential and opposite in SKOV-3 and ISOE-80 cell lines.

### Over-expression of ERα and ERβ in SKOV-3 cells

**Figure 3:**
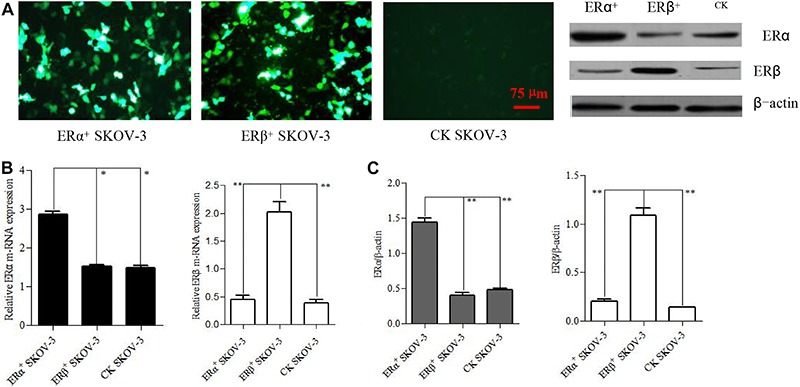
Over-expression of estrogen receptors (ERs) in SKOV-3 human ovarian cancer cells. The 293T pLV-ERα or 293T pLV-ERβ vector co-transfected with SKOV-3 cells was used to generate ERα^+^ and ERβ^+^ SKOV-3 cell lines, respectively. CK SKOV-3 cells referred to blank plasmid transfection. *A*, transfection efficiency validated by inverted fluorescence microscope. Green fluorescence indicates that cells were successfully transfected. *B*, ERs m-RNA expression levels by RT-PCR. *C*, ERs protein expression levels by western blotting. β-actin was used as the internal standard. Data are reported as means±SD (*P<0.05, **P<0.01, ANOVA).

To verify the effect of ERα and ERβ on sema 4D expression, we manipulated over-expression of ERα and ERβ in SKOV-3 using vector construction and lentivirus transfection to generate ERα^+^ and ERβ^+^ SKOV-3 cell lines, respectively. Transfection efficiency was monitored with the inverted fluorescence microscope during the construction of cell lines. The two SKOV-3 cell lines transected with 293T-pLV-ERα and 293T-pLV-ERβ displayed green fluorescence (Figure 3A). The CK SKOV-3 cell line co-transfected with blank plasmid showed no fluorescence under the inverted fluorescence microscope (Figure 3A). Counting analysis indicated that more than 75% SKOV-3 cells were successfully transfected by 293T-pLV-ERα or 293T-pLV-ERβ. After 72 h transfection and culture, RT-PCR and western blotting were conducted to analyze the ERα and ERβ expression level in ERα^+^, ERβ^+^, and CK SKOV-3 cell lines. The results of RT-PCR showed the ERα m-RNA expression level in ERα^+^ SKOV-3 cell lines was significantly higher than in ERβ^+^ and CK SKOV-3 cell lines (Figure 3B). ERα protein expression in ERα^+^ SKOV-3 was also significantly higher than in ERβ^+^ and CK SKOV-3 cell lines by the western blotting analysis (Figure 3C). However, ERβ levels in ERα^+^ SKOV-3 did not exhibit significant differences compared with those in CK SKOV-3 cells. Over-expression of ERα in SKOV-3 cells did not alter ERβ expression. The up-regulation of ERβ in ERβ^+^ SKOV-3 cell line was also verified by western blotting and RT-PCR (Figure 3B and C). Since ERα m-RNA and protein levels in ERβ^+^ SKOV-3 cells were not significantly different from those in CK SKOV-3 cells, the ERβ over-expression did not affect the expression of ERα as well.

### Over-expression of ERα and ERβ conversely regulated sema 4D expression and cell proliferation

**Figure 4:**
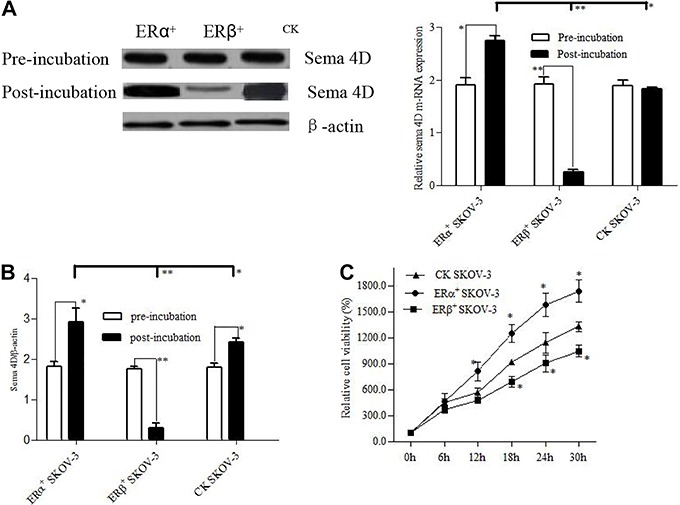
Effect of estrogen receptors (ERs) activator with 17β-estradiol (E2) incubation on semaphorin (sema) 4D expression and cell proliferation. *A*, sema 4D m-RNA expression by RT PCR. *B*, sema 4D protein expression by western blotting. β-actin was used as the internal standard. *C*, Cell proliferation assay. Data are reported as relative viability of cells represented by the ratio of OD_450_ values. Experiments were carried out in triplicate. Data are reported as means±SD (*P<0.05, **P<0.01, ANOVA).

As members of ligand-dependent transcription factor, ERα and ERβ exert transcriptional regulation on the targets only in the presence of a co-factor. E2 hormone is the most common and natural co-factor of ERα and ERβ in the ovary. To initiate ERα and ERβ activity on transcriptional regulation, a 10**^-^** ^6^ M E2 solution was incubated with cells for 6 h, and then followed by analysis of sema 4D expression using western blotting and RT-PCR. In ERα^+^ SKOV-3 cells, sema 4D m-RNA level was increased by 44.2% after E2 incubation (Figure 4A). And sema 4D protein levels were also enhanced by 38.8% in ERα^+^ SKOV-3 cells via E2 incubation (Figure 4B). However, the expressive pattern of sema 4D in ERβ^+^ SKOV-3 was completely different from those in ERα^+^ SKOV-3 cells. The sema 4D expressions in m-RNA and protein levels were found to be reduced by 85.3 and 86.4 % in ERβ^+^ SKOV-3 cells with E2 incubation, respectively (Figure 4A and B). Although sema 4D mRNA levels were similar pre-incubation and post-incubation of E2 in CK SKOV-3 cells, the protein expression of sema 4D was significantly increased with E2 incubation, which was possibly due to the higher expression of ERα than those of ERβ in CK SKOV-3 cells. The cell proliferation of ERα^+^ SKOV-3 was significantly higher than those of ERβ^+^ SKOV-3 and CK SKOV-3 cells starting from 12 h of E2 incubation. The cell proliferation of ERβ^+^ SKOV-3 cells was the lowest (Figure 4C).

### ERα and ERβ regulation on sema 4D expression and cell proliferation was disabled by inhibitor of estrogen signaling pathway

**Figure 5:**
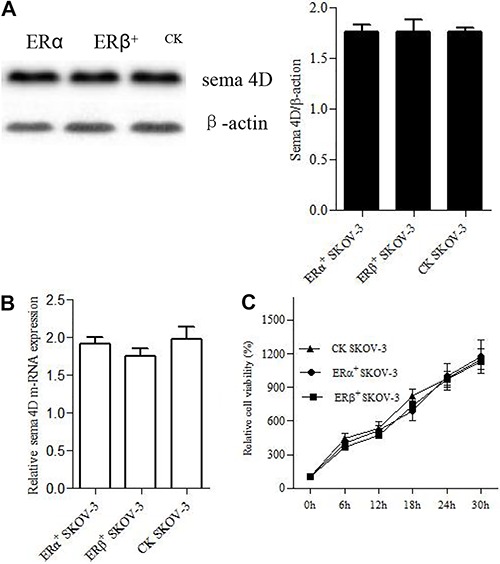
Effect of estrogen receptors (ERs) inhibitor ICI182-780 on semaphorin (sema) 4D expression and cell proliferation. *A*, sema 4D protein expression by western blotting; *B*, sema 4D m-RNA expression by RT PCR. β-actin was used as the internal standard; *C*, cell proliferation assay. Data are reported as means±SD of relative viability of cells represented by the ratio of OD_450_ values.

To confirm that E2 pre-incubation regulated sema 4D expression through ERα and ERβ, an inhibitor of estrogen signal pathway, ICI182-780, which can inhibit both ERα and ERβ activity, was pre-incubated with cells and then followed by E2 treatment and detection of sema 4D expression. The western blotting results showed that the levels of sema 4D proteins in both ERα^+^ and ERβ^+^ SKOV-3 cell lines were recovered to control levels (Figure 5A). And the m-RNA levels of sema 4D in ERα^+^ and ERβ^+^ SKOV-3 cell lines were also parallel to those in CK SKOV-3 cells (Figure 5B). The regulatory effect of ERα and ERβ on the sema 4D expression was completely abolished due to inactivation of ERα and ERβ by ICI182-780 inhibitor and the cell proliferation of the three cell lines was found to be at the same level during the E2 incubation (Figure 5C).

## Discussion

Sema 4D has been reported to be up-regulated in several cancers (20), advancing cancer progress. In the current study, we analyzed sema 4D expression at the cell and tissue levels. Sema 4D was found to be negatively expressed (40/60, **–**) or expressed in a relatively low rate of 33.3% (18/60, +; 2/60, ++) in normal ovarian tissue and patients with ovarian benign tumors had a sema 4D expression rate of 58.3% (18/60, +; 14/60, ++; 3/60, +++). However, only 8.3% (5/60, **–**) of patients with ovarian malignancy negatively expressed sema 4D at an early pathological stage and patients at a late pathological stage showed higher sema 4D protein levels (2/30, +; 12/30, ++; 16/30, +++) than those in an early stage (5/30, -; 4/30, +; 10/30, ++; 11/30, +++). Sema 4D expression was positively correlated with the progress of ovarian cancer. Likewise, we also found that sema 4D m-RNA and protein expression levels in ovarian cancer SKOV-3 cells were significantly higher than those in ISOE-80. A previous study by our group has reported that sema 4D expression was gradually enhanced with the progress of ovarian malignancy using immunohistochemistry. However, the association of sema 4D expression with the ovarian cancer grade was not well assured due to the small sample size used previously. Therefore, an expanded sample size of ovarian malignancy was utilized in the current study. Conclusively, the data further confirmed that sema 4D played an inductive factor in ovarian cancer as they function in other malignancies (27).

ERα expression levels were significantly higher than ERβ in SKOV-3 cells. On the contrary, IESO-80 cells showed a significantly higher ERβ levels than those of ERα in the present study. These observations confirmed the fact that ERα is generally an oncogene and ERβ is an anti-oncogene (28,29). Since sema 4D, ERα, and ERβ expression levels were altered in SKOV-3 cells, we postulated that sema 4D was possibly one of the targets regulated by ERα and ERβ. An over-expression of ERα and ERβ in SKOV-3 cells was manipulated to produce ERα^+^ and ERβ^+^ SKOV-3 cells. ERα and ERβ did not directly affect the behavior of the cancer cells. They function by regulating a variety of targets in cancer cells in the presence of ligands. The two cell lines as well as control SKOV-3 cells were then incubated with natural ERs activator E2 and followed by detection of sema 4D expression. Q-RT PCR results showed that sema 4D m-RNA expression was enhanced in ERα^+^ SKOV-3 cells when treated with E2 solution. Correspondingly, the sema 4D protein was increased in ERα^+^ SKOV-3 cells. As expected, sema 4D expression levels were significantly decreased in ERβ^+^ SKOV-3 via E2 incubation. When cells were pre-incubated with ERs inhibitor followed by E2 incubation, sema 4D expression was not altered. The regulation of ERα and ERβ on sema 4D expression was completely abolished due to inhibition of ERs activity. In addition to regulation of sema 4D expression by ERα and ERβ, we also found that the cell proliferation of E2-incubated ERα^+^ SKOV-3 cells was faster than those of E2-incubated ERβ^+^ and CK SKOV-3 cells. The E2-incubated ERβ^+^ SKOV-3 cells multiplied even slower than CK SKOV-3 cells. Sema 4D up-regulation was directly attributed to the faster proliferation. The fact was further validated by pre-incubation of *ICI182-780* inhibitor, which can remove the differentiation of both sema 4D expression and cell proliferation of E2-incubated ERα^+^, ERβ^+^ and CK SKOV-3 cell lines. The results suggested that oncogene property of ERα was possibly due to its positive regulation on sema 4D expression and acceleration of cell multiplication, whereas ERβ negatively controlled sema 4D expression and inhibited cell proliferation.

ERα and ERβ have been proven to show similar affinity to natural 17β-estradiol, which is the main female hormone secreted in ovary (30). Excluding the other factors that induce ovarian cancers, the risk of natural hormone utilization on the ovarian cancer may depend more on the ERα/ERβ ratio. ERα and ERβ have an approximate ratio of 1/2 in normal ovarian tissues, therefore, women who did not accept steroid-resembling estrogen treatment had a lower risk of having ovarian cancer (31). However, when women used steroid-resembling estrogen drugs in MHT, the risk of ovarian cancer was carefully evaluated because synthesized estrogen analogues possibly bind to ERα and ERβ unequally (32,33). Based on our data, the use of steroid-resembling estrogen analogues in MHT, which have a higher affinity to ERα than ERβ, would increase the risk of ovarian cancer by up-regulating sema 4D expression and accelerating the multiplication of ovarian cells. The steroid estrogen drugs with a lower affinity to ERα were more favorable in MHT to reduce the occurrence of ovarian cancer. The ERα/ERβ ratio rather than the numbers of ERα and ERβ isoforms, is a more useful marker in the diagnosis of ovarian cancer for the women who did not accept MHT beforehand.

In conclusion, the data presented here suggested that sema 4D was up-regulated and was an inductor in the progress of ovarian cancer. Sema 4D expression and cell proliferation of ovarian cancer was first proven to be regulated by ERα and ERβ in an opposite manner. Although the regulatory mechanism of ERs on sema 4D expression remains vague, the clues might help peers to explore it. A cautious use of estrogen drugs in MHT is advised.
